# Bilateral choroidal metastasis of follicular thyroid carcinoma 7 years after total thyroidectomy: a case report

**DOI:** 10.5935/0004-2749.2022-0055

**Published:** 2024-02-23

**Authors:** Theo R. Morais, Nicole B. Larivoir, Cristiana M. Ramalho

**Affiliations:** 1 Faculdade de Medicina, Centro Universitário de Volta Redonda, Volta Redonda, RJ, Brazil; 2 Universidade Federal de Juiz de Fora, Juiz de Fora, MG, Brazil; 3 Oftalmo Centro de Oftalmologia, Juiz de Fora, MG, Brazil

**Keywords:** Choroid neoplasms/secondary, Neoplasm metastasis, Adenocarcinoma, follicular, Thyroid neoplasms

## Abstract

Follicular thyroid cancers account for 15%-20% of all thyroid tumors. Choroidal metastases secondary to follicular thyroid cancer rarely occur. Herein, we report the case of an 85-year-old woman who presented choroidal metastasis from a follicular thyroid carcinoma in the right eye 7 years after total thyroidectomy and underwent enucleation. To confirm the diagnosis and primary tumor site, histopathological, and immunohistochemical examinations were performed. One year later, she presented metastasis in the contralateral eye. Few similar cases have been described in the literature.

## INTRODUCTION

The incidence of thyroid cancer has continuously and globally increased in recent decades, making it the ninth most common malignant tumor. Despite these alarming demographic data, distant hematogenous spread to the uvea is still exceptionally rare, corresponding to only 0.5%-6% of uveal metastases^(1,2)^.

Herein, we report a case of bilateral uveal metastases secondary to follicular thyroid carcinoma. To the best of our knowledge, only nine cases of choroidal metastasis of follicular thyroid carcinoma have been described in the literature^(2-9)^, and only two of these cases were bilateral^(3,6)^.

## CASE REPORT

**Figure 1:**
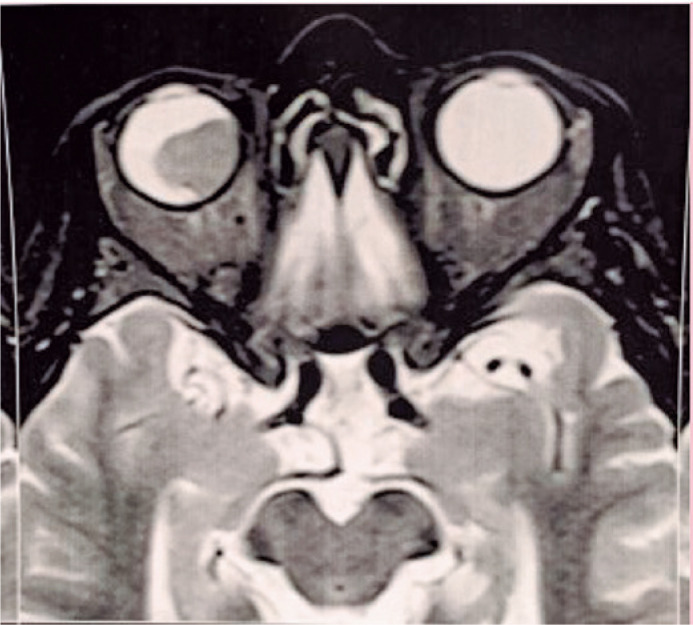
Magnetic resonance imaging showing intraocular mass.

**Figure 2:**
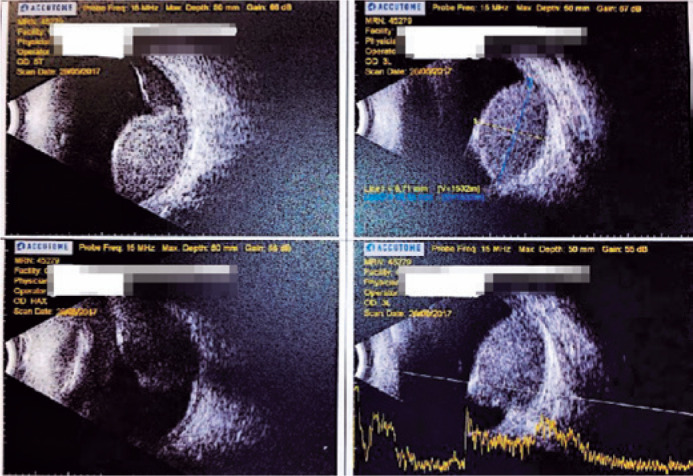
Ultrasonography: elevation with medium internal echogenicity and hyper-echoic points; serous inferior retinal detachment.

**Figure 3:**
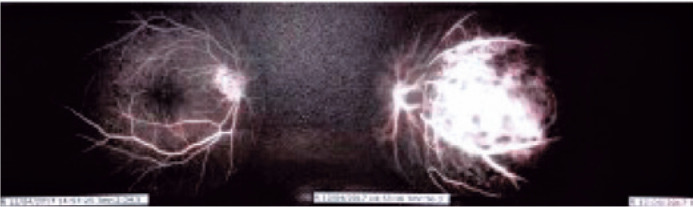
Retinography: nasal retinal elevation with dotted fluorescence and physiological vasculature.

An 85-year-old Caucasian woman was referred in March 2017 for consultation after a fine-needle biopsy in an elevated choroidal tumor in the right eye (OD), by an outside ophthalmologist. The lesion was diagnosed following a complaint of 2-month unilateral progressive vision loss. Ocular magnetic resonance imaging and ultrasonography before the procedure demonstrated a single choroidal mass with associated serous retinal detachment (Figures 1 and 2), and fluorescein angiography showed a nasal choroidal mass (Figure 3).

During the first evaluation, she complained of severe eye pain. The best-corrected visual acuity (BCVA) was no light perception (NLP) in OD and 20/30 in the left eye (OS). Tonometry of applanation measurements were 30 mmHg in the OD and 14 mmHg in the OS. The examination of the ocular adnexa revealed eyelid hematoma and edema, with total ptosis, and exophthalmos in the OD. Biomicroscopy showed total hyphema, hematic corneal infiltration, corneal edema, and nonvisualization of the pupil and iris in the OD. Findings on slit-lamp and dilated fundus examinations were within the normal limits in the OS.

The patient’s medical history was significant for thyroid carcinoma in June 2010. She had undergone total thyroidectomy, and pathological examination identified extensively invasive slightly differentiated thyroid follicular carcinoma. Moreover, she had received two courses of radioactive iodine 131 therapy in January 2014.

Owing to the intense pain, NLP visual acuity, and result of the ocular pathological examination of the fine-needle biopsy of positive smears for neoplastic cells, enucleation of the right globe was preferred. The pathology of the enucleated globe confirmed extensive necrotic carcinoma in the choroid with infiltration of the vitreous body and retina. Immunohistochemical examination revealed a probable thyroid origin, which evolved with complete improvement of pain, good hea-ling, and adaptation of esthetic scleral prosthesis. The patient was then referred to the oncology department that diagnosed her with brain and backbone metastases and started treatment with radiotherapy.

In December 2018, she returned complaining of worsening vision in the OS, in which her BCVA was 20/50. Findings on slit-lamp examination and applanation tonometry measurements were within normal limits. A dilated fundus examination revealed an elevated macular mass. An ocular sonogram showed a choroidal mass with irregularly high internal reflectivity, with a basal diameter of 11 mm and thickness of 4 mm. Angiofluoresceinography identified a hyperfluorescent elevated macular mass. As the diagnosis was compatible with ocular metastasis, the family, and oncologist decided not to pursue any ocular intervention. The patient died 6 months after the diagnosis of metastasis in the second eye.

## DISCUSSION

The most common types of thyroid carcinoma are papillary (70%), follicular (15%), anaplastic (5%), and medullary (3%) thyroid carcinomas^(1)^.

Follicular thyroid carcinoma has its peak incidence between the fifth and sixth decades of life, predominantly among women^(3)^. The usual mode of spread is hematogenous dissemination, with the lungs and bones as the most common sites of metastases^(3)^. Choroidal metastasis is rare and occurs more frequently in patients with advanced disease portending a poor prognosis^(9)^.

Despite its rarity, follicular thyroid carcinoma must be considered in the differential diagnosis of a uveal mass of unknown origin, especially in patients with a disease history, even if the primary tumor was diagnosed many years ago. Considering choroidal metastases from follicular thyroid carcinoma described in the literature, only three cases had ocular symptoms as the initial disease manifestation^(3,6,9)^. In other cases, the time between thyroid carcinoma diagnosis and ocular symptoms varied between 1 month and 40 years (mean 13.5; median 7 years)^(2,4,5,7,8,9)^.

Choroidal metastasis can be asymptomatic^(7)^ or present with various symptoms. Decreased vision is the most common, especially if the metastasis involves the optic nerve or macula^(2-5,8-9)^. Cases associated with an overlying retinal detachment can present with flashing lights and floaters^(1)^.

Effective treatment options for thyroid carcinoma metastasizing to the eye include external-beam radiation, radioactive iodine 131 therapy, and brachy--radiotherapy using a 125I episcleral radioactive plaque insertion. Enucleation is the treatment of choice for patients with metastases that cause a definitive vision loss and/or persistent pain^(1)^.
